# A neural network-based exploratory learning and motor planning system for co-robots

**DOI:** 10.3389/fnbot.2015.00007

**Published:** 2015-07-23

**Authors:** Byron V. Galbraith, Frank H. Guenther, Massimiliano Versace

**Affiliations:** ^1^Program in Cognitive and Neural Systems, Boston UniversityBoston, MA, USA; ^2^Center for Excellence in Learning in Education, Science, and Technology, Boston UniversityBoston, MA, USA; ^3^Neuromorphics Laboratory, Boston UniversityBoston, MA, USA; ^4^Department of Speech, Language, and Hearing Sciences, Boston UniversityBoston, MA, USA; ^5^Department of Biomedical Engineering, Boston UniversityBoston, MA, USA

**Keywords:** co-robot, exploratory learning, motor planning, neural network, egocentric navigation, embodied AI

## Abstract

Collaborative robots, or co-robots, are semi-autonomous robotic agents designed to work alongside humans in shared workspaces. To be effective, co-robots require the ability to respond and adapt to dynamic scenarios encountered in natural environments. One way to achieve this is through exploratory learning, or “learning by doing,” an unsupervised method in which co-robots are able to build an internal model for motor planning and coordination based on real-time sensory inputs. In this paper, we present an adaptive neural network-based system for co-robot control that employs exploratory learning to achieve the coordinated motor planning needed to navigate toward, reach for, and grasp distant objects. To validate this system we used the 11-degrees-of-freedom RoPro Calliope mobile robot. Through motor babbling of its wheels and arm, the Calliope learned how to relate visual and proprioceptive information to achieve hand-eye-body coordination. By continually evaluating sensory inputs and externally provided goal directives, the Calliope was then able to autonomously select the appropriate wheel and joint velocities needed to perform its assigned task, such as following a moving target or retrieving an indicated object.

## Introduction

Co-robots, collaborative robots that work alongside humans to perform assistive tasks, are becoming more prevalent, notably in the healthcare and telepresence spaces (Kristoffersson et al., 2013). A major challenge for co-robots is the need to make decisions on how to operate in dynamic environments with other autonomous agents (Hayes and Scassellati, 2013). This includes using onboard sensors to detect and avoid obstacles or finding, reaching for, and grasping objects. Embodying the co-robot with some sense of spatial awareness is critical for it to make appropriate decisions on how to proceed with its tasks.

Spatial awareness here refers to the combination of sensory inputs, such as visual and proprioceptive, to construct an egocentric coordinate system for objects in the immediate vicinity of the co-robot. The sensory processing, decision-making, and motor planning components of the task process all share this reference frame in order to achieve effective coordination. For instance, the co-robot needs to know where its body and arm are relative to a visually identified target object in order to plan and execute the appropriate motor actions needed to achieve its goal of grasping the object. If the robot is too far away to reach for a target object from its current position, it will have to move its body closer until the target is within range.

A common first step in developing co-robot control models is to employ simulations and virtual environments to evaluate which strategies and methods have a chance of working in the real world. By avoiding issues such as battery charge and wear and tear of robot parts in simulations, multiple models can be evaluated rapidly without fear of damage to physical components. The main drawback to relying on virtual environments is that many challenges faced in the real world are difficult to simulate accurately without significant effort. Perfectly aligned idealized components of a robotic limb in the virtual environment will have isotropic movement behavior, while in the real world, compliance in the mounting joint and inconsistent servo performance will result in anisotropic movements. Even more challenging is the reliance on data from actual sensors, which are susceptible to noise and artifacts, whereas simulated models frequently use perfect information and highly constrained environments.

These variances between idealized models and physical reality may not be describable analytically, which poses a significant challenge in translating theoretical control systems to practical application. One solution is to embody the co-robot with an adaptive system that integrates and learns actual sensory and behavioral data. By using exploratory learning methods, the robotic agent is able to use a form of unsupervised learning where it gains an operational model of its capabilities by observing the results of its own actions. As the co-robot performs and observes the results of endogenous random movements, i.e., motor babbling, it learns how to link sensory information with motor actions. Once these causal relationship models are built, the co-robot can then transition from passively observing undirected actions to actively planning goal-directed actions.

In this work we present such a system using an adaptive neural network-based controller that employs exploratory learning to enable a hardware robot to autonomously search for, navigate toward, and pick up a distant object as specified by a remote operator. In order to evaluate the viability of the learning, sensory integration, and decision-making models required for these tasks in both virtual and hardware versions of the Calliope robot, we created the CoCoRo (Cognitive Co-Robot) control system. Using CoCoRo, we demonstrate that through motor babbling of its wheels and arm, the Calliope is able to learn how to relate visual and proprioceptive information to achieve the hand-eye-body coordination required to complete its intended tasks.

The rest of this paper is arranged in the following way. Section Materials and Methods describes the CoCoRo architecture, the Calliope robotic platform used to evaluate the system, and a detailed description of the components used to achieve hand-eye-body coordination. Section Results presents the results of several experiments conducted to validate the reaching, navigation, and distant object retrieval goals. In Section Discussion, the methods and experimental results are discussed and compared to previous work. The paper concludes in Section Conclusion with a summary of the key contributions.

## Materials and methods

### CoCoRo architecture

**Figure 1:**
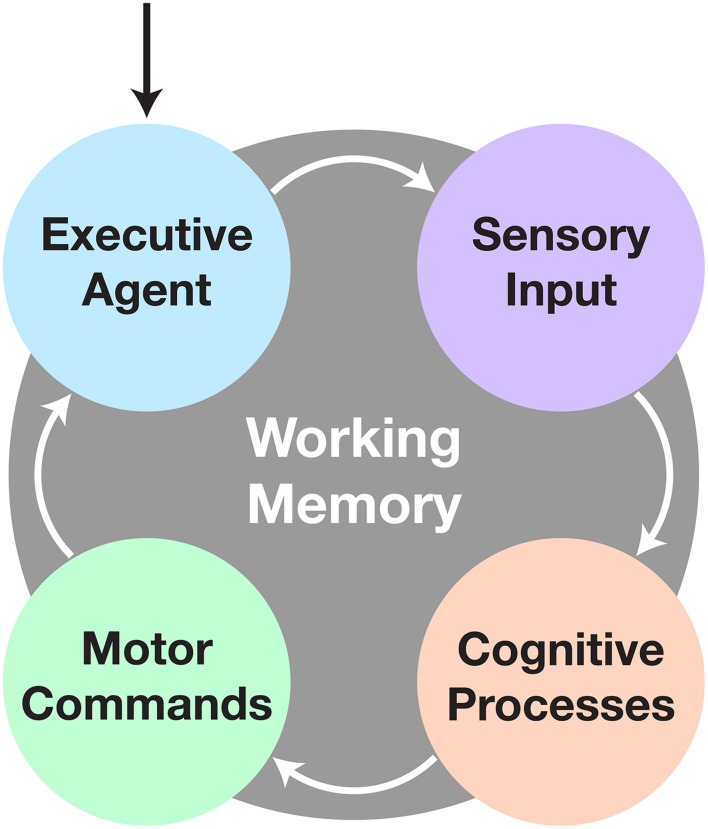
**The cognitive cycle**. After initialization, the cognitive cycle runs until the user halts the robot. The executive agent checks for changes in goal directive, followed by acquisition of sensory data. Next comes processing of the data to fulfill the current objective. Finally any new motor commands are sent to the appropriate devices and the process repeats. All communication during and persistence across cycles is handled by the working memory system.

CoCoRo uses a modular, synchronous architecture. It defines four types of system components: executive agent, sensorimotor devices, cognitive processes, and working memory. Each component in the system is chained together in serial with data flowing from one component to the next via a data structure termed a cognitive packet. A single iteration through all components is referred to as a cognitive cycle (Figure 1).

The cognitive cycle consists of four phases: executive, sensory, cognitive, and motor. In the executive phase, a cognitive packet is generated by the working memory component, which includes persistent information from the last cycle, time elapsed since the beginning of the previous cycle, and any commands from the executive agent. Next, in the sensory phase, all sensorimotor devices are polled to retrieve new raw sensory data. Then, in the cognitive phase, cognitive processes act on the sensory and memory data. Finally, in the motor phase, the sensorimotor devices execute any relevant motor commands generated from the previous phases. Finally, the executive agent is given the opportunity to store or transmit any data from the cognitive packet before the next one is generated and the cycle repeats.

The executive agent determines the broad goal objective and task the co-robot will perform. This could arise endogenously through a default behavior pattern or exogenously through commands received from a remote operator. The executive agent also has the ability to store or transmit data for later analysis or telepresence capabilities. Sensorimotor devices are elements that produce sensory data and/or execute motor commands, such as capturing image data from a camera or setting velocity commands to wheel motors. Cognitive processes are intended to be discrete, single purpose functions, such as detecting objects in a visual scene or planning the motor actions needed to articulate a limb toward a desired target. These processes operate on either raw sensory data or the outputs of upstream processes. They then either output intermediate data for use by downstream processes or drive behavior in the form of motor commands. Finally, working memory retains persistent information over the duration of the designated task operation, such as what the goal target is, where it was last seen, and whether certain actions should be enabled or inhibited.

**Figure 2:**
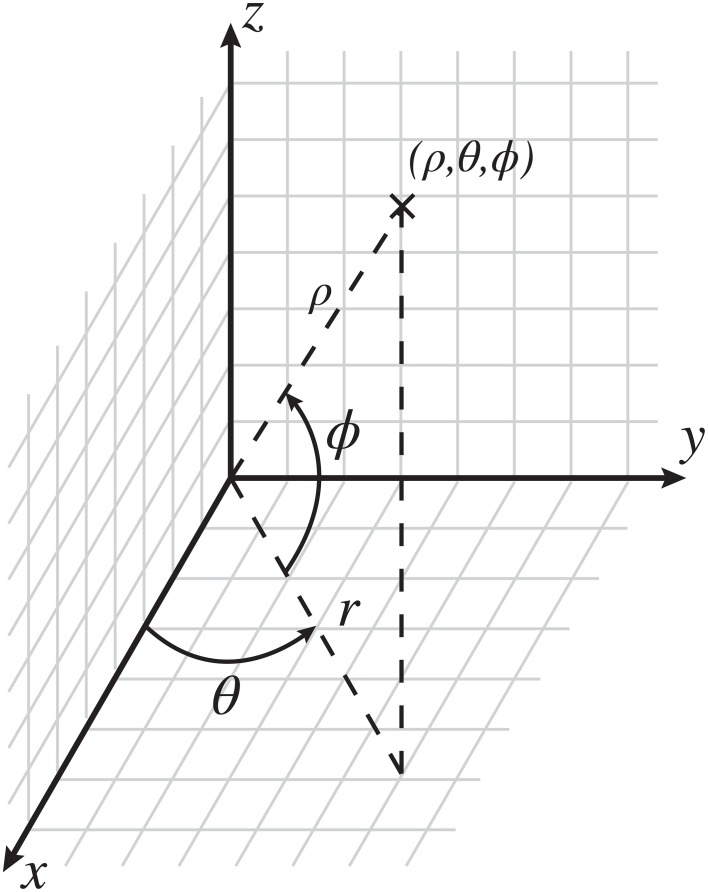
**The CoCoRo common coordinate reference frame**. The origin is defined as the center of the robot's head. In Cartesian space, *x* is in front of the robot, with positive values going outward, *y* is the horizontal plane, with positive values going to the left, and *z* is the vertical plane, with positive values going up. In spherical space, ϱ is the distance from the origin to a given point, θ is the counterclockwise azimuth angle in radians, and ϕ is the inclination angle upward from the horizontal plane in radians.

The CoCoRo architecture separates out the realization of a specific robotic platform from the cognitive control model by defining an API for writing the robot control system component modules and runtime programs. Using this approach, cognitive processes evaluated in a virtual environment can be directly applied to a real world robot without code changes—only the CoCoRo runtime, including operational parameters, and the sensorimotor device modules need be specific to a particular robot environment. Additionally, a common reference frame for working with various coordinate systems in three dimensions is also defined as part of this API to ensure consistent operation between components (Figure 2). All code was implemented using the Python programming language.

### Robot platform

**Figure 3:**
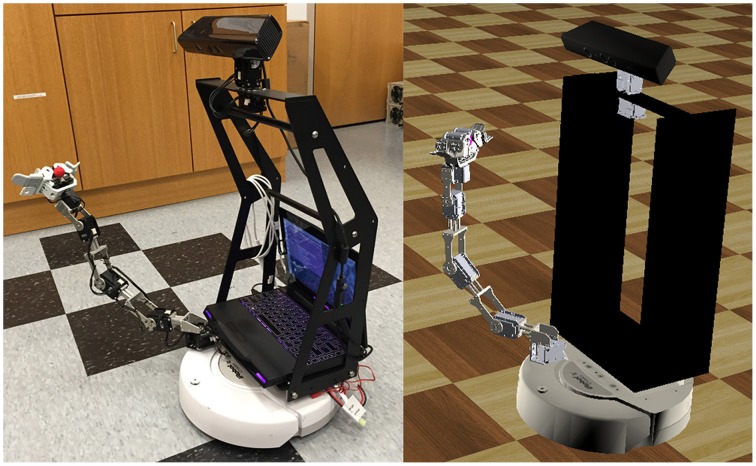
**The Calliope robot**. The RoPro Calliope mobile robot (left) and its virtual counterpart in the Webots (http://www.cyberbotics.com/) robotics simulator (right).

The robot platform used in this study is the RoPro Calliope (Figure 3), a reference robot designed for the Tekkotsu robotics development environment (Tira-Thompson and Touretzky, 2011). The Calliope is a multimodal system consisting of an iRobot Create robot base mounted with a 7-degree-of-freedom (DOF) robotic limb and a Microsoft Kinect. All hardware components of the Calliope are centrally controlled via a laptop running Linux (Ubuntu 14.04) resting on top of the Create.

The Create is a differential-drive robot with two drive wheels capable of up to 500 mm/s either forward or reverse and a third balancing wheel. The limb is constructed from Robotis Dynamixel servos and separated into a 4-DOF arm with horizontal shoulder, vertical shoulder, elbow, and wrist pitch joints on one servo network and a 3-DOF hand with wrist roll and two claws on another network. Each servo has 1024 addressable positions covering 300 degrees. The servos are controlled through a USB-to-TTL interface. The Kinect has a 640 × 480 32-bit color camera and a 640 × 480 12-bit depth camera. The cameras have a field of view of 1 radian horizontal and 0.75 radians vertical. The depth camera has an effective sensing range of 0.5–3.5 m. Pan and tilt control of the Kinect is provided by two additional Dynamixel servos also on the arm servo network. Power for the Kinect and arm servo network comes from a battery pack mounted on the back of the Create, while the hand servo network is powered from the Create's own battery. When fully assembled, the Calliope weighs 10.34 kg.

To enable safe testing and evaluation of the CoCoRo control system and component modules, a virtual representation of the Calliope was developed in Webots (Michel, 2004), a commercial mobile robot simulation software package. Webots allows for robot controllers to be written in a variety of languages including Python, which made it ideal for testing and evaluating the various CoCoRo components.

### System implementation

On top of the base CoCoRo platform we developed the components necessary to embody the Calliope with the ability to reach and grasp distant, visually identified objects. This task required the co-robot to perform the following coordination of subtasks: identify and localize objects in the environment, visually search for a desired object, navigate toward the object, reach for the object, and finally grasp the object in its hand.

**Figure 4:**
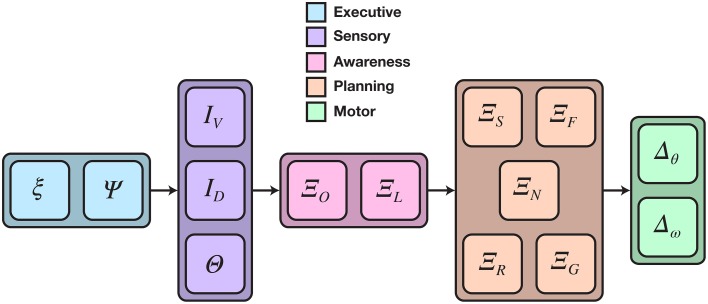
**Detailed cognitive cycle model for reaching and grasping distant objects**. Data flows from left to right. Vertically aligned components could execute in parallel, though in practice all components execute in a single serial chain. The cognitive process phase was divided into two sub-phases: object awareness and motor planning. ξ is the cognitive packet, Ψ is the executive agent, *I* and Θ are data from camera and joint position sensors, respectively, Ξ is a cognitive process, and Δ is a motor command expressed as a joint or wheel velocity.

The CoCoRo components created to fulfill this task include an executive agent that supported remote operator control; four sensorimotor device interfaces for the Calliope's Kinect, servos, and wheels; and multiple cognitive processes to perform decision-making and coordination for the various subtasks. The full cognitive cycle implementation is depicted in Figure 4.

The executive agent was implemented using the Asimov middleware system (Galbraith et al., 2011) to send and receive data between the Calliope and a remote operator. Operators were able to send goal directives and manual motor commands. They could also optionally receive video frames from the Calliope's camera. Additionally, the agent had the capability to store the contents of each cognitive packet to disk after the end of a cycle for later offline analysis.

Four sensorimotor devices were created: one for the Kinect, one for the Create, and one for each of the two servo networks. The Kinect device captured and provided the raw RGB and depth images while the Create device accepted and issued changes in wheel velocity. The servo devices, corresponding to the arm/neck and hand servo networks, provided the current positions of the joints, set the joint velocities and goal positions, and translated between CoCoRo's common reference frame and the internal Dynamixel reference frame.

The cognitive processes were divided into two functional groups: object awareness and motor planning. Object awareness consisted of two steps: detecting known objects in the visual scene and then localizing them in reference to the body. Motor planning contained the processes for generating and coordinating joint and wheel velocities to control head position, navigation, reaching, and grasping.

As employing robust computer vision methods to object detection was outside the scope of this work, we intentionally chose a simplistic approach. The robot used a color threshold method to detect predefined objects in a constrained environment. Objects were monochromatic cylinders and spheres defined by channel ranges in the CIELAB color space. CIELAB was chosen over RGB due to its greater robustness to changes in luminance. First the raw RGB image was converted to CIELAB using OpenCV and then segmented into a 5 × 5 grid of tiles. For each known object, the tile with the most matching pixels that fell into that object's color range was selected. The object was considered present if the pixel count exceeded a threshold of 64 pixels. The centroid of the object was then computed by taking the median x and y image coordinate values of all matching pixels. The depth value was selected by taking the corresponding pixel location from the depth image. Finally, these pixel values were added to the cognitive packet along with the object's label.

Object localization converted all detected objects from raw image coordinates (*I*_*x*_, *I*_*y*_, *I*_*z*_) into relative egocentric locations (ρ,θ,Φ). The angular coordinates of each object were computed using the following transforms:

(1) $$\theta \;=\left(\frac{1}{2}-\frac{I_{x}}{I_{w}}\right)F_{h}+\theta_{p}$$

(2) $$\Phi \;=\left(\frac{1}{2}-\frac{I_{y}}{I_{h}}\right)F_{v}+\theta_{t}$$

Here, *I*_*w*_ and *I*_*h*_ were the image width and height in pixels, *F*_*h*_ and *F*_*v*_ were the horizontal and vertical fields-of-view, and θ_*p*_ and θ_*t*_ were the positions of the pan and tilt joints. This had the effect of converting raw pixel locations into retinotopic coordinates and then adjusting them based on the head position.

For the Kinect, *I*_*z*_ ranged from 0 to 2047, with 0 corresponding to >3.5 m, 2046 corresponding to approximately 0.5 m, and 2047 corresponding to an error code meaning no depth information was obtained. If an error code was detected, no value was set for ρ, otherwise it was computed by:

(3) $$\rho =D\left(I_{z}\right)+l_{n}sin\left(-\theta_{t}\right)$$

The first part transformed the Kinect pixel values to depths given in meters using function *D* adapted from Miller (2010). The second part adjusted for the tilt of the head away from center, where *l*_*n*_ = 0.05 m was the length of the neck.

Once objects were detected and localized, they were passed on to the motor planning processes. Head position was determined by whether or not the goal object was detected in the visual scene. When the target was not detected, joint commands were generated to rotate the head in a fixed sweeping pattern to scan the environment until the target was found. Otherwise the robot fixated on the target by generating joint commands to position the head such that the target was held in the center of vision. For the scope of this work, no additional seeking behavior was implemented, so the robot remained stationary while scanning the environment indefinitely if the target could not be detected.

#### Reaching

Motor planning for reaching is based on the DIRECT model (Bullock et al., 1993; Guenther and Micci Barreca, 1997), which belongs to the class of psuedoinverse control methods for redundant manipulators (Klein and Huang, 1983). These methods solve the inverse kinematics problem of choosing appropriate joint velocities that achieve desired end-effector movement by computing the generalized psuedoinverse of the manipulator's Jacobian matrix.

**Figure 5:**
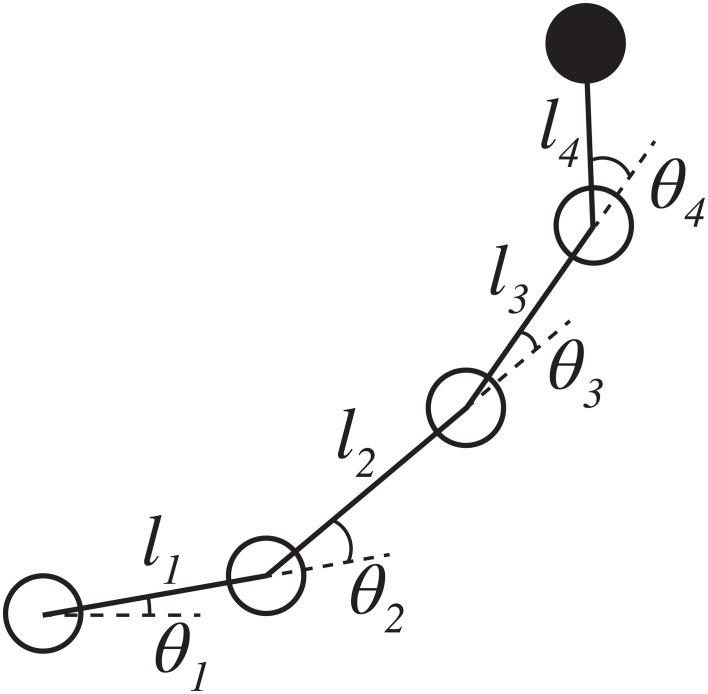
**Stick model of the Calliope arm**. The Calliope arm has four revolute joints arranged in a linear chain. The first joint represents horizontal shoulder movement and rotates about the *z*-axis. The other three joints, vertical shoulder, elbow, and wrist pitch, respectively, rotate about the *y*-axis. The limb segment lengths are 0.11, 0.145, 0.138, and 0.135 m, respectively.

There are two challenges to implementing this solution in practice. First is that the Jacobian matrix must be computable for all possible joint configurations. In stick models or simulations where the robot is treated as a rigid body and the exact geometry of the arm is known, the solution can be computed directly. For instance, the Calliope's limb (Figure 5) has the following ideal relationship between joint configuration and end effector's egocentric location:

(4) $$\begin{array}{l} x_{e}=x_{0}+\cos\theta_{1}(l_{1}+l_{2}\cos\theta_{2}+l_{3}\cos(\theta_{2}+\theta_{3})\\ \;+\;l_{4}\cos(\theta_{2}+\theta_{3}+\theta_{4})) \end{array}$$

(5) $$\begin{array}{l} y_{e}=y_{0}+\sin\theta_{1}(l_{1}+l_{2}cos\theta_{2}+l_{3}\cos(\theta_{2}+\theta_{3})\\ \;+\;l_{4}\cos(\theta_{2}+\theta_{3}+\theta_{4})) \end{array}$$

(6) $$\begin{array}{l} z_{e}=z_{0}+l_{2}\sin(\theta_{2})+l_{3}\sin(\theta_{2}+\theta_{3})\\ \;+\;l_{4}\sin(\theta_{2}+\theta_{3}+\theta_{4}) \end{array}$$

where (*x*_0_, *y*_0_, *z*_0_) is the location of the base of the arm in the CoCoRo common reference frame, *l*_*i*_ is the length of the *ith* arm segment, and θ_*i*_ is the position of the *ith* joint. Using this, the Jacobian matrix and psuedoinverse can be easily derived and computed.

In the real world, however, the Calliope is susceptible to deviations from this model due to the invalidation of the rigid body assumption, operational limitations, and minor manufacturing defects. As such the error between the actual and computed Jacobian will vary in an inconsistent fashion across the workspace. This is further compounded by the second challenge to using the inverse kinematic model, which is determining where the hand is relative to the desired location.

Obtaining the value for the desired end-effector displacement, Δ*x*, in a simulation could be as straightforward as tracking the allocentric coordinates of both end effector and desired target and then computing their difference. In an embodied system, where the robot can only act upon data from its sensors, arriving at an appropriate value for Δ*x* is non-trivial. The desired reach target is located through the visual system, whereas the hand can be located through vision or, failing that, through an estimate achieved via proprioception. This latter modality is especially important, as the robot's hand may not be visible when reaching is initiated toward a target. Good hand-eye coordination, i.e., agreement between visual and proprioceptive position estimates, is important for obtaining consistent values of Δ*x* and thus for maintaining smooth and effective reaching trajectories.

DIRECT addresses both the determination of the Jacobian and achieving good hand-eye coordination through neural network-based exploratory learning mechanisms. By motor babbling the joints in the arm and observing the resulting position of the end effector, the DIRECT neural network is able to learn the relationship between the visual and proprioceptive inputs. Using this method accounts for deviations from the idealized model by using actual data instead of theoretical predictions.

Our version of DIRECT is similar to that described in Guenther and Micci Barreca (1997) as we also use a hyperplane radial basis function (RBF) network (Stokbro et al., 1990; Du and Swamy, 2014) as our choice of neural network. However, we do not attempt to learn the inverse map, but instead only learn the forward map and then use it to numerically approximate the instantaneous Jacobian matrix. This is accomplished by querying the trained model for expected changes in end effector position due to slight perturbations of each joint in isolation. Once obtained, the arm joint motor plan is computed using the psuedoinverse method.

In addition to learning how to articulate its limb to reach for a particular location, the robot also needs to determine if that location is actually within its immediate reach, a task outside the scope of the DIRECT model. We have developed a solution to this reachability problem using the same motor babbling process employed by DIRECT. The reachability of a desired object is whether or not the robot can move its end effector to that exact location from its current position. Both the geometry of the robot's arm and the persistent features of its operational environment determine the reachable workspace of the robot, such as the robot's own body morphology and the relative position of the floor. An object is labeled as reachable if it is contained within a manifold encompassing all points that the end effector can move through. Defining this manifold is not achievable through simple polyhedral, however. Every place the hand can go is considered a reachable location; therefore all recorded locations of the hand are collected into a point cloud that represents a sampling of the reachability manifold. A Delaunay triangulation, a mesh of adjacent simplices, is then constructed from this set of points, which creates a convex approximation of the manifold. Additionally, like the RBF network, the Delaunay triangulation algorithm supports incremental update allowing it to be used in both offline and online learning scenarios. The test for reachability of an object becomes whether or not its location would fall within the boundaries of any simplex in the mesh. When a goal object is outside the range of reachability, the navigation system is disinhibited allowing wheel commands to be generated to move the robot toward the target as described in the next subsection. As soon as the object is deemed to be within reachable range, the navigation system is inhibited, preventing any further wheel movements.

Limited grasping capabilities were also implemented. For the purposes of this work, the actual grasping problem was reduced from 3DOF to 1DOF by making all grasping targets vertically aligned cylinders, e.g., soda cans. The wrist pose never had to change as it was always aligned for vertical targets, and the finger and thumb motor actions were treated as one synchronous motion to jointly open or close. The distance vector between the location of the hand and the target object that was computed during the reaching task was evaluated each cycle against a minimum grasping threshold. Once the hand was determined to be within this threshold for grasping the target, motor commands were issued to both close the hand at a fixed velocity and cease any new reaching-related joint velocities.

#### Motor babbling

Motor babbling is an exploratory-based learning strategy for sensorimotor control. Through repeated execution of the action-perception cycle, an agent is able to build an internal model of how its motor behavior corresponds to sensory observations. The babbling aspect is that random actions are generated to explore and discover the range of possible outcomes with limited or no prior knowledge of what is actually possible. This strategy has been successfully used in neural network-based embodied learning for navigation (Zalama et al., 1995) and reaching (Bullock et al., 1993) using endogenously generated pseudorandom joint velocities. A drawback of those approaches, however, is that there is no active exploration of the workspace. Instead they passively rely on a large number of trials to fully cover the space. Recent approaches have explored an active form of motor babbling that either uses a confidence metric in accuracy to direct babbling to less confident regions (Saegusa et al., 2009) or a curiosity-driven reinforcement learning method that seeks out unexplored regions (Frank et al., 2014).

For this work, a semi-active approach was utilized. Endogenous random joint or wheel velocities were generated as in the passive case, but Sobol sequences (Sobol, 1976) were used instead of uniformly distributed pseudorandom numbers. A Sobol sequence is a set of quasi-random numbers designed to evenly cover a space for given sequence length. This provides a semi-active solution, as although it is still largely random, it is guaranteed that the babbling phase will result in actions that explore the entire workspace, thus reducing the number of training iterations required.

#### Navigation

The Calliope, owing to the iRobot Create base, uses a differential drive form of locomotion. Like with reaching, in order to navigate toward a desired target, the robot needs to solve the inverse kinematics problem of determining the wheel velocities that will move it to the appropriate location. Typically solved in allocentric, Cartesian space (Dudek and Jenkin, 2010), we present an egocentric, polar space solution that produces smooth trajectories.

**Figure 6:**
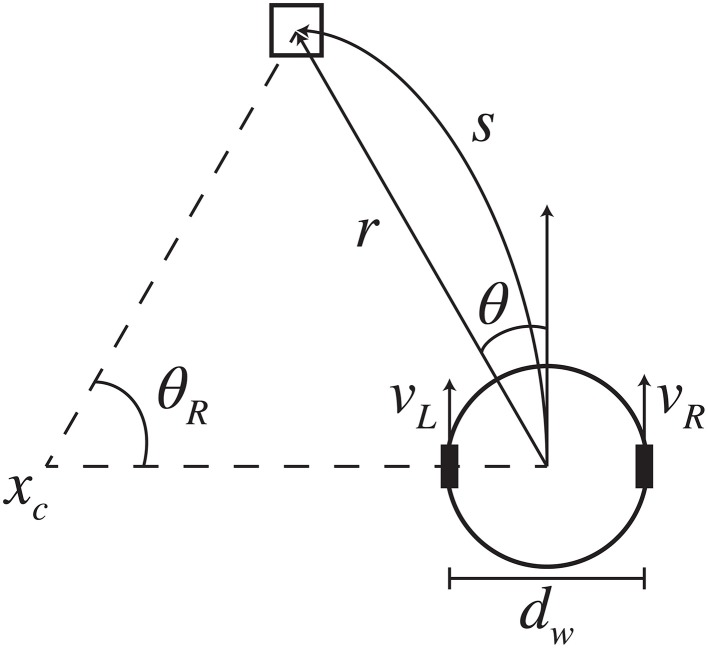
**Differential-drive kinematic model**. Based on the visually determined relative location of the desired target (*r*, θ), the robot generated wheel velocities (*v*_*L*_, *v*_*R*_) to produce the trajectory arc that would reach the target. The arc has length *s* and angle of rotation θ_*R*_ about point *x*_*c*_.

Assuming constant wheel velocities (***v***_*R*_, ***v***_*L*_) with no slippage over a fixed time interval, the inverse kinematic model is initially given as:

(7) $$\left[\begin{array}{c} v_{R}\\ v_{L} \end{array}\right]\Delta t=\left[\begin{array}{cc} 1 & \frac{d_{w}}{2}\\ 1 & -\frac{d_{w}}{2} \end{array}\right]\left[\begin{array}{c} s\\ \theta_{R} \end{array}\right]$$

where *d*_*w*_ is the distance between the wheels and ***s*** is the desired trajectory arc length with angle of rotation θ_*R*_. Determining (*s*, θ_*R*_) is challenging when working in allocentric coordinates, where the robot must have a sense of the target location and its own relative to a fixed origin in the environment. This problem is avoided when working in egocentric coordinates, where the robot views everything in relationship to itself (Figure 6). The relationship between egocentric coordinates in the horizontal plane (*r*, θ) and the associated trajectory arc is:

(8) $$s=\frac{\theta r}{sin\theta }$$

(9) $$\theta_{R}=2\theta$$

By combing Equations (7–9) the egocentric inverse kinematics model is obtained:

(10) $$v_{R}\Delta t=\left(\frac{\theta r}{sin\theta }+\;\theta d_{w}\right)$$

(11) $$v_{L}\Delta t=\left(\frac{\theta r}{sin\theta }-\;\theta d_{w}\right)$$

In practice, however, the wheel velocities have maximum speeds (*v*_*Rmax*_, *v*_*Lmax*_) that this model does not accommodate; simply capping or scaling velocities that exceed these limits is insufficient as the difference between *v*_*R*_ and *v*_*L*_ is central to the desired trajectory movement and must be preserved. Let Δ*t* = 1 s, *v*_*Rmax*_ = *v*_*Lmax*_= *v*_*max*_, and:

(12) $$\delta =\frac{v_{R}-v_{L}}{2}=\theta d_{w}$$

then considering the imposed requirement of non-negative velocities, the wheel velocities are given by:

(13) $$v_{R}=\max\left(\min\left(\frac{\theta r}{sin\theta },v_{max}\right)-\left|\delta \right|+\delta ,0\right)$$

(14) $$v_{L}=\max\left(\min\left(\frac{\theta r}{sin\theta },v_{max}\right)-\left|\delta \right|-\delta ,0\right)$$

In egocentric space, the relative position of the target is continually changing while the robot is moving, so new velocities are generated every cycle. As no distinction needs to be made between stationary and moving targets as long as they can be localized, this method can produce smooth trajectories for both approaching a fixed location and pursuing a mobile object.

## Results

**Figure 7:**
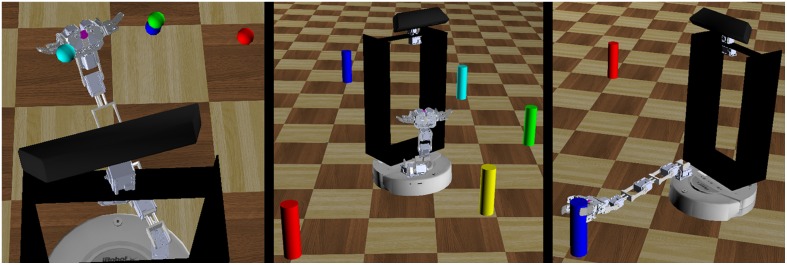
**Three robot behavioral experiments**. The robot performed a series of behavioral tasks to evaluate the feasibility of the motor babbling approach. These tasks included repeatedly reaching to a series of targets in space (left), navigating toward a target and stopping within a set distance threshold (center), and grasping distant objects (right).

The hand-eye-body coordination tasks were evaluated in three broad task areas: hand-eye coordination, egocentric navigation, and grasping distant objects (Figure 7). These experiments were conducted in both virtual and real world environments.

### Hand-eye coordination

The co-robot performed arm motor babbling to learn both the relationship between proprioceptive inputs of joint positions to the visual inputs of end-effector position and an approximation of the reachability manifold of the arm. Random target joint positions were generated over [−2.62, 2.62] radians per joint with velocities chosen to require 10 cognitive cycles to reach the new position. During this motor babbling phase, the co-robot fixated on its hand, identified by either a magenta circle (virtual) or red foam ball (real) attached to the end effector. If the end effector was visually located during a cognitive cycle, the arm joint positions and target location were recorded.

After the motor babbling phase ended, an offline training phase was conducted. Data outliers due to noise from the real world cobot were identified and rejected by detecting target positions with a nearest neighbor distance greater than 2.5 cm. A Delaunay triangulation was constructed from this data to approximate the reachability manifold.

A hyperplane RBF network was trained to learn the forward proprioceptive map. First a grid search was conducted using the collected data to determine the number of bases, Gaussian width, and learning rate to use for the network—the Gaussian centers were spread evenly across the joint input space of [−2.62, 2.62] radians per joint. Next, 10,000 distinct evenly spaced joint configurations and associate hand positions were generated from the rigid-body model of the arm (Equations 4–6) and used to prime the network. Finally, the network was trained on the collected data. To imitate online learning, data points were presented sequentially and only once.

The network parameters chosen for both virtual and real world cobot were three bases per input dimension for a total of 3^4^ or 81 bases, σ = 1.57, and α = 0.025. The network trained from within the virtual environment was able to reproduce the training set target positions with *R*^2^ = 0.926 and RMSE = 0.051 while the network trained on the real world Calliope achieved *R*^2^ = 0.942 and RMSE = 0.044.

**Figure 8:**
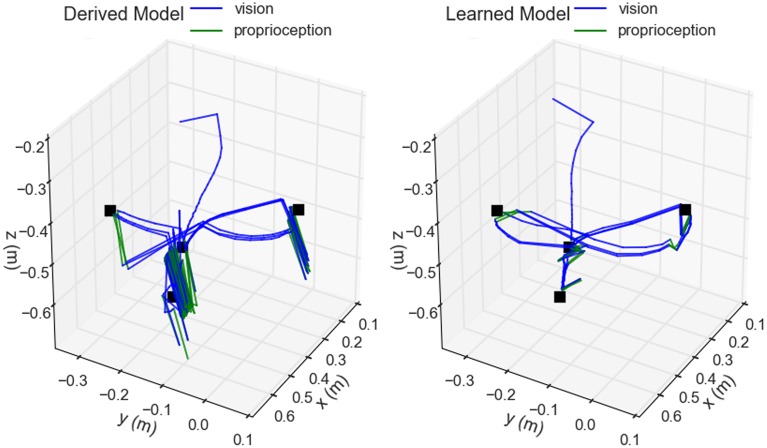
**Comparison of derived vs. learned models for hand-eye coordination**. The trajectory of the hand positions as determined by the robot are shown during the execution of a reaching task cycling between four visually located targets (black). Blue components of the trace indicate when the hand was visually located, whereas green indicates when the proprioceptive model was used. Arm joint velocities were determined using Jacobian matrices either computed directly from the rigid-body model (left) or approximated from the trained neural network (right).

The efficacy of the hand-eye coordination model acquired through motor babbling was then compared to that of one based on the rigid-body model. The virtual co-robot reached toward four colored targets suspended in the space in front of it in a predetermined order. The hand was deemed to have reached the target if the difference between detected positions was within (0.02, 0.034, 0.034) spherical units. Once reached, the co-robot moved to the next target in the sequence, completing the entire cycle three times. The position of the hand as determined by the robot was recorded and plotted (Figure 8).

### Egocentric navigation

Motor babbling of the wheels allowed the robot to learn the distance between its wheels. It fixated on a target initially placed 1.5 m directly in front of it, recorded the target's position provided from the visual system, then engaged each wheel at a fixed velocity selected from a Sobol sequence over [−0.15, 0.15] m/s for approximately 1 s. After the trial time had elapsed, the robot came to a halt, recorded the new relative position of the target, and computed the wheel distance estimate using:

(15) $$\bar{d_{w}}=\frac{\Delta t}{\Delta \theta }(v_{r}-v_{l})$$

**Figure 9:**
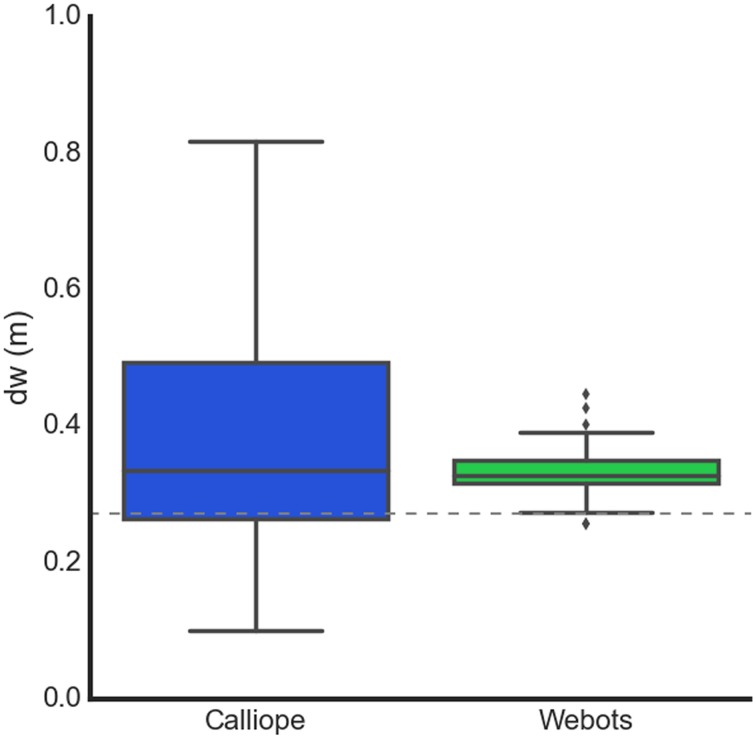
**Learning body size through motor babbling**. The Calliope learned a *d*_*w*_ of 0.336 ± 0.088 m (*n* = 91), while the virtual robot learned a *d*_*w*_ of 0.326 ± 0.014 m (*n* = 84). The dotted line at 0.272 m represents the actual distance between the wheels.

It repeated this process using the reverse of the previously selected velocities to return to its approximate starting position. After several trials of forward and reverse pairs were conducted, the median of the estimates was taken as the robot's learned wheel distance (Figure 9).

Using the learned wheel distance, the robot navigated toward targets placed approximately 1 m away and at −90°, −45°, 0°, 45°, and 90° angles. The robot stopped once it determined it was within 20 cm of the target. Once the robot stopped moving, the actual distance between the edge of the target and the center of the robot was measured and recorded. The real and virtual robots achieved mean stopping distances of 22.7 ± 0.748 cm (*n* = 15) and 20.2 ± 0.458 cm (*n* = 5), respectively.

**Figure 10:**
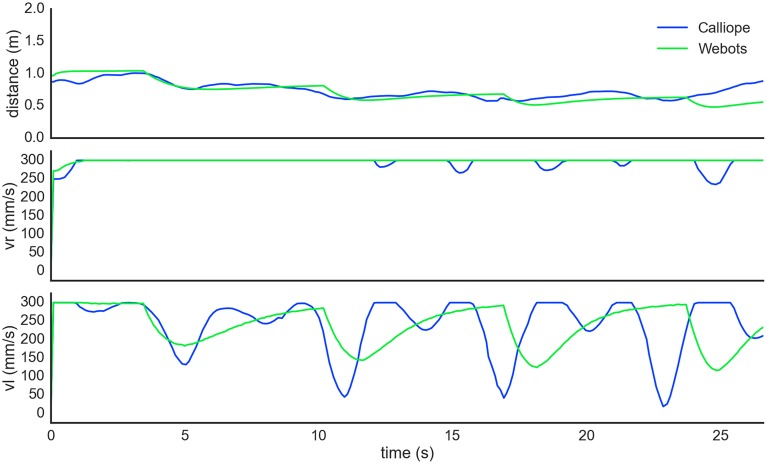
**Autonomous pursuit task**. The robot visually tracked and pursued a target moving counterclockwise in a square pattern. The self-determined distance between the robot and target (top) slowly decreased as the robot got closer during the turns. Right wheel velocity (center) was kept at maximum while left wheel velocity (bottom) modulated during turns. The dips in both the right and left wheel velocities of the Calliope (blue) following a corner turn are from the robot overshooting and correcting itself.

To demonstrate an example of human-robot interaction, the robot also followed a person identified by a held target object. The person started 1 m directly in front of the robot, holding the identifying object approximately 0.7 m off the ground. The person then walked in an 8 m perimeter square pattern just fast enough to prevent the robot from catching up. This was replicated in the virtual environment by having the target object hover above the ground and move on its own. During this task, the position of the target was smoothed using an exponential weighted moving average to mitigate sensor noise. Both the virtual and real world robots maintained pursuit over traversal of the pattern (Figure 10).

### Grasping distant objects

**Figure 11:**
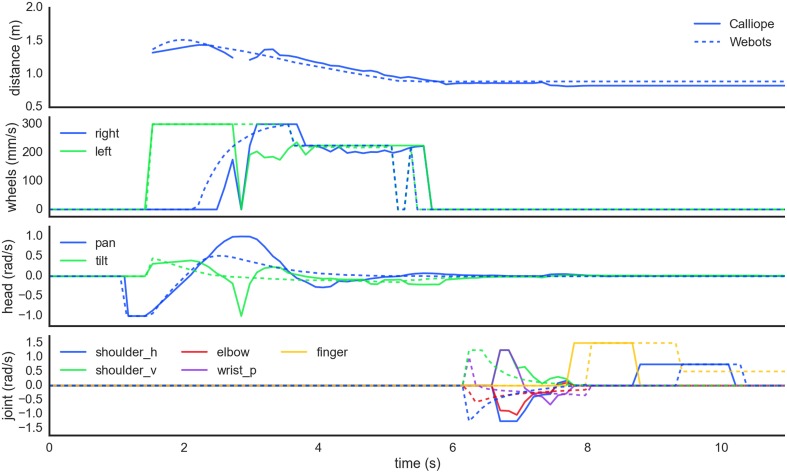
**Motor planning coordination while picking up a distant object**. In order from the top, these plots show the detected distance to the target object followed by the generated wheel velocity, head position, and limb joint commands, respectively for both real (solid) and virtual (dashed) robots. First the robots scan the scene searching for the target. At 1.5 s, they locate the target to the right and navigate toward it while maintaining gaze fixation. Around the 5.5 s mark, the robots determine the object is reachable, stop navigation, and ensure head position is stable before starting to reach toward the target. Grasping is initiated around 8 s in and takes about 1.5 s to complete before the obtained target is finally lifted off the ground.

The coordination of reaching and navigation was demonstrated in a task where the Calliope had to pick up an operator-directed target in the environment. The Calliope was placed in an environment with two (real) or three (virtual) known objects located at (1.5 m, 0°), (1.4 m, −45°), and (1 m, 45°) away, all outside the immediate grasping range of its arm. It was then activated and assigned one of the objects to find and pick up. The robot had to coordinate head position, wheel velocities, and arm and hand joint velocities to complete the task successfully (Figure 11). The virtual robot performed one trial for each target and managed to grasp and lift each for 100% completion. The real robot performed five trials for each target and successfully completed the task 80%, 40%, and 60% of the time, respectively, for an overall completion rate of 60%. In all cases where the Calliope failed to complete the task, it was because it grazed the target object with its hand, knocking it over. It still managed to stop within reaching distance and move its hand to the correct vicinity of the target. Videos of both virtual and real robots performing the task can be found in the Supplementary Materials.

## Discussion

### The CoCoRo control system

One of the design choices with CoCoRo was to use a serial, synchronous data flow model. This was chosen for its relative simplicity of implementation and the ability to chain certain cognitive processes together in a defined order for coordination purposes. However, the penalty for using this architecture was that the entire cognitive cycle was rate-limited by the slowest component. This had no impact on the virtual environment where simulation time had no bearing on real time, but it did affect the real robot, where the object identification process proved slowest due to the naïve implementation of color matching applied to the relatively large input image. Many other robot platforms, including Tekkotsu and MoBeE (Frank et al., 2012), use threaded, finite state machine architectures, which can achieve real-time performance and take advantage of concurrent and distributed processing of information. This avoids the rate-limiting problem of the serial architecture at the cost of increased system complexity. However, with the computational power inherent in modern laptops, like the one mounted on the Calliope, CoCoRo's simplistic structure did not interfere with the ability of the robot to complete tasks effectively. The Calliope operated at an average rate of 10 Hz during task execution, which was sufficiently fast enough to adjust motor commands as needed for the tasks undertaken albeit with the maximum wheel and joint velocities artificially reduced. Wheel velocities were capped at 300 mm/s and arm joint velocities were capped at ±1.5 rad/s. The simulation step time in Webots was set at the default value of 32 ms. As all sensor and motor component control steps must be a multiple of this simulation step, 96 ms was chosen to offer a comparable decision performance rate.

**Figure 12:**
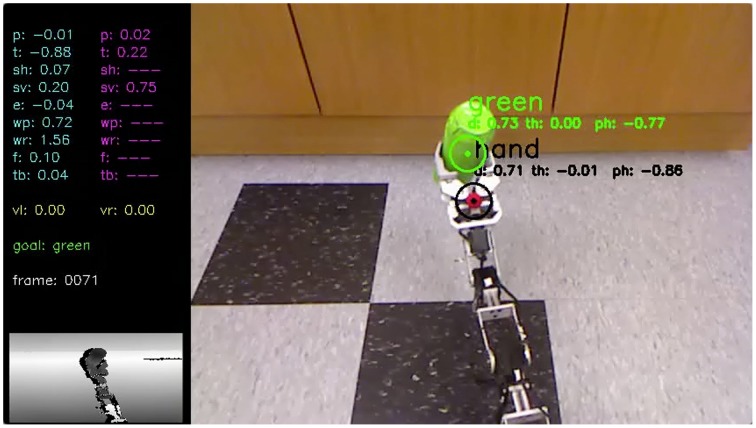
**Calliope lifting an object**. This is a frame taken from a movie (see Supplementary Materials) reconstructing the Calliope's point of view during a task to grasp and lift a green object initially located 1.5 m away. The movie is created from stored cognitive packets generated during the execution of the task and includes all sensory inputs, motor commands, and identified objects.

An additional benefit of using the serialized data flow model was the ability to easily capture and store the cognitive packet to disk, the data structure that contained all the sensory inputs, intermediate processing, and motor outputs from a given time point. This process was used extensively for both debugging purposes and offline analysis, such as providing the data for several of the figures in this paper. A tool was also created to reproduce robot point-of-view movies from these packets (Figure 12), which proved invaluable for tracking down issues with object detection and localization.

### Virtual environments

The use of simulations and virtual environments are key to developing and evaluating robotic control systems. If the virtual environment provides a good enough approximation of the real environment, certain tasks can be bootstrapped in the simulation first, such as building up the internal neural network weights for control tasks. These weights can then be transferred directly to the physical co-robot which would then need a shorter recalibration learning session than if it had started with untrained networks. We used just such a method in training the neural network responsible for reaching. Training it first using an idealized set of inputs to outputs primed the network and provided reasonable results for locations in the reaching space that were not obtainable through motor babbling alone, i.e., where vision failed to detect the hand. The later data collected from motor babbling was then able to retrain the network to be more in line with the actual observed results instead of those generated by the rigid-body approximation.

However, we also encountered several discrepancies when moving between virtual and real sensors. The images from the virtual Kinect were always crisply rendered, whereas the images being pulled from the real Kinect were susceptible to noise. The sources of noise included motion blur introduced by movement from the body and head, and potential changes in luminance due to automatic white balancing performed by the Kinect video camera. The depth camera in the virtual environment, like the virtual video camera, was generated from the OpenGL buffer directly and did not suffer the effect of infrared shadows. These shadows were areas visible in the video image but in which no depth information could be obtained due to objects in the foreground preventing the infrared signals from reaching them. Despite these challenges in using video and depth image data in the real environment, the Calliope was still able to perform at a high level for the tasks explored, though additional checks had to be added for cases in which objects were visible but no depth information could be obtained.

Likewise, the behavior of servos varied between simulation and reality. In the virtual environment, servos would move smoothly in response to any requested velocity within defined operational range and supported high precision positional accuracy. The real servos, on the other hand, were limited by having only 1024 addressable positions for a resolution of about 0.005 radians. This contributed to occasional jittery behavior when attempting to hold joints in a particular pose due to the effects of rounding. The real servos also did not support specifying a velocity of zero to halt movement. Instead, we had to rely on a combination of velocity and positional control to achieve a fixed joint configuration. Finally, the skeleton of the arm itself contained screws prone to loosening during continual operation, resulting in slight changes to the position of the end effector over time.

### Hand-eye coordination

Controlling redundant joint manipulators is an open challenge in robotics, as closed-form analytic solutions to the inverse kinematics problem may not exist. Feedback-based control strategies have proven successful, but require reasonably accurate sensors to provide the needed error signals. These can be difficult to acquire for a non-planar limb outside of simulation or highly controlled workspaces. As a requirement of co-robots is to operate in largely uncontrolled environments, the control system should not rely on external sensors and fixed workspaces. We used a variant of the DIRECT model, a biologically inspired neural network approach to feedback-based control of a limb. Desirable features of DIRECT that make it useful for co-robots are that it is egocentric, so all sensor information comes from its own perspective, and it can adapt to changes in limb configuration. However, DIRECT, like many other solutions, was validated in simulation using perfect knowledge of end-effector position and stick-model limbs. Other applications of DIRECT have been reported (Vilaplana and Coronado, 2006; Grosse-Wentrup and Contreras-Vidal, 2007; Bouganis and Shanahan, 2010), but these too were only performed in simulation with perfect positional knowledge and lack of physical constraints beyond joint rotation boundaries. Our implementation is the first instance we are aware of that demonstrates the efficacy of DIRECT using actual computer vision to determine end-effector and target localization. Furthermore, this is also the first demonstration of DIRECT embodied in a real-world robot working in a 3D workspace.

Using visual inputs from a camera and working with a physical robot presented its own set of challenges for DIRECT, computer vision not with standing. DIRECT uses motor babbling to learn the space of movements, so it must be able to observe the end-effector in order to learn how it moves in a particular part of the workspace. With a fixed camera vantage point, body components obstructing views, and limitations of the camera sensor, the Calliope had several blind spots. Our solution to this was to prime the network before motor babbling commenced using the rigid-body model of the arm to generate thousands of training points evenly spaced across the entire hypothetical workspace. The network was trained using a learning rate an order of magnitude lower than that used during motor babbling so that real observed data would take precedence.

**Figure 13:**
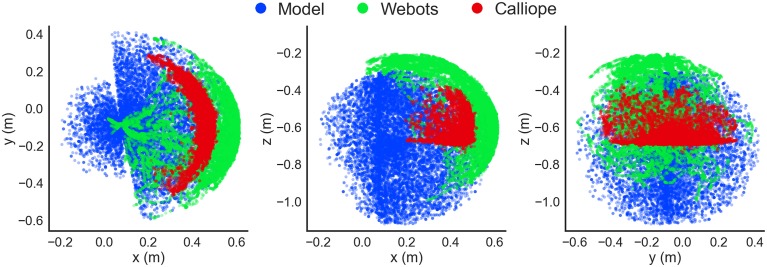
**Detected hand position during motor babbling**. Recorded hand positions are shown in the xy- (left), xz- (center), and yz-planes (right). A kinematic stick model (blue) computed hand positions using randomly generated joint positions and the geometry of the arm, while both the Webots virtual environment simulation (green) and Calliope (red) used visual information to determine hand position during a motor babbling task.

For instance, the observed location of the robot's hand in the virtual environment displayed close similarity to that of the rigid-body model for the portion of the workspace the arm was able to reach during motor babbling (Figure 13). As can be seen, this actually represented only a fraction of the theoretical range if the arm was free of any obstacles. The use of an identifying color marker on the top of the hand also produced a compressed range of visible locations. If the configuration on the arm resulted in the hand positioned upside down, for instance, it would not be recognized.

The major difference between theoretical and detected position came in the real world Calliope, where the detected distance of the hand was almost 10 cm on average closer than the model would predict. This can be attributed to two factors: a greater offset from the location of the visual marker to the end of the hand and the less precise distance estimation from the actual Kinect's depth camera vs. the simulated Kinect. The observed range of motion for the real hand was even more compressed than the virtual one, however, due to the Kinect's blindness within close proximity. Relying solely on either observed data or theoretical model would have produced large gaps or erroneous estimates, respectively. Using the theoretical model for initially priming the RBF neural network then further training with the motor babbling results provided a solution that enabled the use of both approaches to complement each other.

For actually generating joint trajectories during a reach task, the analytically determined Jacobian from the rigid-body model produced similar behavior to that approximated by the trained neural network in three of the four reaching segments. The rigid-body model, based in Cartesian coordinates, produced straighter trajectories between targets but had significant disagreement between its visual and proprioceptive locations as exhibited by the trajectory shifts when switching between the modalities occurred. This most impacted the model during the downward trajectory from target three to four, where it got stuck and convulsed for several seconds before finally achieving a correct configuration. This was due to the first target, placed just above and in front of the fourth, occluding the marker on the hand toward the end of the trajectory resulting in the model flipping between visual and proprioceptive locations. The disagreement between the two was large enough that, when using visual input, the hand was perceived where it actually was, above the target, but when using proprioception, the hand was perceived to be below the target. This conflict produced the observed spasms. While all targets were eventually reached here, in a separate instance the arm became locked into a never-ending cycle of jittering up and down and the trial had to be terminated. The neural network model, by contrast, was based in spherical coordinates, produced slightly arced trajectories, and had much greater agreement between proprioception and vision. It experienced no difficulties in any of the reaching segments. Even when losing sight of the hand, there was enough agreement in the two modalities to allow for consistent smooth behavior during the trials.

### Egocentric navigation

In determining wheel distance, the real and virtual robots produced very similar final estimates, with the main difference being the noisiness of the Calliope's samples. Both robots were over the actual distance by 6 and 5 cm, respectively. This error could be related to the relative distances between wheels, camera, and reference target, as extending the wheel distance out further in the virtual environment produced very accurate estimates. This error did not appear to have an impact on the actual navigation tasks, as both the stationary and pursuit tasks produced comparable results. In the stationary task, the difference in average stopping distance was only 2.5 cm, while in the pursuit task, the Calliope performed well despite slightly overshooting the turns then having to correct.

This method for egocentric navigation employs an aiming strategy (Franz and Mallot, 2000) for local navigation, where the goal of path planning is to keep a desired target position directly in front of the robot while moving toward it. Other aiming approaches include Concentric Spatial Maps (CSM) (Chao and Dyer, 1999), which uses a neural network to store goal positions and obstacles in discrete locations arranged in concentric circles around the agent. A similar, though non-neural, approach to CSM is used to produce multi-agent pedestrian navigation through crowds (Kapadia et al., 2012). Both of these methods account for obstacles whereas we assumed a clear path. CSM, however, requires the environment map be loaded *a priori*, while the pedestrian model does not use sensory information from the agents themselves and instead determines them from the global simulation state.

An alternative and complementary strategy to aiming is guidance (Franz and Mallot, 2000), where the relative positions of environmental cues are used to determine desired trajectories. Examples of guidance-based approaches include ENav and variants (Altun and Koku, 2005; Fleming, 2005). They are based on the sensory egosphere (SES) (Albus, 1991), a 2D spherical projection of incoming sensory data to a spatial representation of the agent's environment, where the goal is to match the angular displacements of visually identified landmarks in the current SES with those provided in the desired SES. ENav is the only other method we are aware of to have reported implementation attempts outside of simulation (Fleming, 2005), though with limited results.

These navigation methods provide path planning abstracted from a specific kinematic model of locomotion. While ostensibly more general, they may produce trajectories that are not possible by an actual mobile robot, so an appreciation for the inverse kinematics of locomotion for target robot platforms is critical to produce a model that can work in real environments.

For differential-drive navigation, the inverse kinematics problem can be solved by breaking down the desired trajectories into pairs of distinct motions: first rotate in place to face the target, then drive straight forward toward it (Dudek and Jenkin, 2010). This, however, produces jerky motion, requiring the robot to stop forward progress every time it needs to rotate. For a clear path in an ideal environment, the expectation would be only one rotation and one direct forward trajectory. However, in a real world environment, wheel slippage, dynamic target location, and perturbations in the floor can result in deviations from the ideal trajectory, requiring compensatory corrections, each resulting in the robot having to stop, rotate, and begin forward again. This would be especially inefficient in the egocentric model, where the relative positions of objects are always changing as the robot moves.

Similar arc-based solutions to the one above have been proposed in both Cartesian (Bethencourt et al., 2011) and polar (Maulana et al., 2014) forms, though the former relies upon accurate accumulation of encoder data to reconstruct allocentric position while the latter is geared toward following a fixed track. Instead of learning just the body size as demonstrated here, the NETMORC model (Zalama et al., 1995) attempts to learn the inverse kinematic solution itself through a neural network trained via a similar motor babbling phase. However, only simulated results with perfect positional information used in training the network were reported.

This is the first work we are aware of that combines the use of egocentric navigation with a specific model of inverse kinematics. Not only does this approach succeed with a high accuracy in simulation, it works very well in a real world robot despite the increased noise from and limitations of actual hardware and environments.

### Grasping distant objects

The task of grasping and lifting distant objects combines the previously described subtasks into a unified whole, requiring an additional layer of coordination on top of the individual motor plans. The motor planning coordination strategy used in this work was to take a largely lock-step approach, where the individual subtasks were disinhibited only when their role was called upon. The only exception to this was head movement, which operated in parallel to the progression of navigation, reaching, grasping, and lifting. This coordination was implemented by having each cognitive process in the chain alter or check the working memory system and inhibiting or disinhibiting itself based on its state.

Two main factors can be attributed to the cases where the real robot failed to complete the grasping task by knocking the target over. First is the simplistic object identification method, which is highly susceptible to noise and treats objects as points. This results in generally poor performance when precision adjustments were needed, which were typically required due to the second factor, the segregated process of only reaching once navigation stopped. In this arrangement, the arm is held out and to the side until the reaching subtask begins. It makes a downward arcing trajectory to reach the target, which can result in the hand clipping the side of the object if the robot is even a centimeter too close. If the hand began its reach earlier while the robot was still driving forward, the hand could be brought into position before there was a risk of inadvertent contact.

Other approaches to visually guided mobile manipulators employ more fluid motor control and coordination (Andaluz et al., 2012a,b; Kazemi et al., 2012). Related to the co-robot goal of working in unstructured environments, (Xie et al., 2014) presents a model for visual-guided control for grasping household items. All of these systems use a camera mounted on the end-effector instead of elsewhere on the body. These eye-in-hand visual servoing systems can achieve greater grasping and manipulation accuracy at the expense of having to manage a potentially highly articulated neck, i.e., the arm itself, when not engaged in an actual reach action. They also lack the flexibility of the alternate hand-to-eye approach used by the Calliope.

The simplistic method for visual object detection worked well enough for both reaching and navigation in the virtual environment where color detection is much easier. It was less effective in the real world as it was highly susceptible to noise. For navigation, which operated in 2D, this proved less of an issue, but it did impact the success of reaching and grasping, which required accurate 3D locations. The grasping method used was also the simplest available. Real world use would require more intelligent grasping algorithms for shaping the hand to accommodate a variety of object shapes. As CoCoRo supports drop in replacement of components, upgrading to more robust computer vision and grasping processes would be possible.

The egocentric model worked well for traversing the immediate vicinity of the robot assuming a clear path to the target destination. If any obstacles were in its path that did not occlude the target object, however, the robot would attempt to drive through them. Likewise, if the robot failed to detect the desired target in its sensory field, it would either have to revert to an allocentric representation to derive new egocentric coordinates from memory or engage in some form of directed search.

## Conclusion

We presented a control system with an eye toward co-robots that used motor babbling to enable a robot to learn about aspects of its own configuration in regards to hand-eye-body coordination. This system was built on a software platform designed to enable modular evaluation of the learning, sensory processing, and decision-making motor components across both virtual and physical versions of the Calliope robot. The capabilities embodied in the robot enabled it to autonomously follow a person around a room and retrieve distant objects specified by a remote operator. In order to achieve this we demonstrated a variant of the DIRECT neural model for reaching in a hardware robot and complemented it with novel methods for determining if the intended reach target is actually within the robot's grasp and a means for egocentric-based navigation to drive it toward the target if it isn't.

There is still significant work to be done in order to extend this initial system to more practical real-world co-robot use. Adapting to cluttered and dynamic environments would require a much more robust and powerful form of visual object detection and identification that the simplistic model currently used. The navigational system would also be extended to handle obstacle avoidance and combine allocentric and egocentric path planning strategies. Smooth concurrent motor control coordination would also be a desirable improvement over the current lock-step approach.

### Conflict of interest statement

The authors declare that the research was conducted in the absence of any commercial or financial relationships that could be construed as a potential conflict of interest.
